# REFRAMING AGING: PRACTICES AND MEASURES TO HARNESS THE STRENGTH OF INTERGENERATIONAL NETWORK TIES

**DOI:** 10.1093/geroni/igz038.2330

**Published:** 2019-11-08

**Authors:** Shannon E Jarrott, Karen Hooker, Nancy Morrow-Howell

**Affiliations:** 1 The Ohio State University, Columbus, Ohio, United States; 2 Oregon State University, Corvallis, Oregon, United States; 3 Washington University, St. Louis, Missouri, United States

## Abstract

GSA and other agencies have undertaken to “Reframe Aging” to reduce pervasive global ageism, which has been associated with negative attitudes towards and disinterest in working with older adults and poor health in old age. Familial and non-familial intergenerational contact, that is between members of non-adjacent generations, provides valuable opportunities to reduce ageism through relationships. However, much of the research on intergenerational relationships focuses on attitudes, usually those young people hold about older adults and aging. We propose that attention should shift towards measuring the relationship and giving voice to both younger and older partners. Presenters will address a range of intergenerational relationships focusing on their measurement as a means to advance the Reframing Aging initiative, including examples, challenges, and recommendations. Jarrott will provide an overview of the Reframing Aging initiative and address measurement of non-familial intergenerational relationship. This will be complemented by Pillemer’s focus on how universities can reframe aging through intergenerational network ties, including the Cooperative Extension System. Mendoza and Fruhauf will shift attention to familial connections, focusing on grandparent-grandchild caregiving relationships. Turner, Hooker, and Jarrott will present new efforts to develop a validated theory- and evidence-informed measure of intergenerational relationships that can be completed by young and old persons. Discussant Morrow-Howell, past GSA President, will address how a long history of diverse intergenerational solutions can support efforts to reframe aging through enhanced measurement tools and strategies.

